# *Fusarium oxysporum* and *Aspergillus* sp. as Keratinase Producers Using Swine Hair From Agroindustrial Residues

**DOI:** 10.3389/fbioe.2020.00071

**Published:** 2020-02-11

**Authors:** Karina Paula Preczeski, Caroline Dalastra, Fabiane Fernanda Czapela, Simone Kubeneck, Thamarys Scapini, Aline Frumi Camargo, Jessica Zanivan, Charline Bonatto, Fábio Spitza Stefanski, Bruno Venturin, Gislaine Fongaro, Helen Treichel

**Affiliations:** ^1^Laboratory of Microbiology and Bioprocess, Federal University of Fronteira Sul, Erechim, Brazil; ^2^Department of Chemical and Food Engineering, Federal University of Santa Catarina, Florianópolis, Brazil; ^3^Department of Agricultural Science, Agricultural Engineering Post-Graduate Program, Universidade Estadual do Oeste do Paraná, Cascavel, Brazil; ^4^Laboratory of Applied Virology, Federal University of Santa Catarina, Florianópolis, Brazil

**Keywords:** biotechnology process, agroindustry residues, enzyme precipitation, keratinase, swine hair

## Abstract

Technological processes mediated by microorganisms and enzymes are promising alternatives for treatment of recalcitrant residues. Keratinases hydrolyze keratin, the primary component of some wastes generated in many industrial activities. The present study was designed to evaluate strategies for obtaining keratinases produced by fungi using submerged fermentation and two residues as substrates, chicken feathers and swine hair. Two fungi isolated from feather residues showed potential for keratinase production, *Fusarium oxysporum* and *Aspergillus* sp. These were subjected to submerged fermentation using chicken feathers and swine hair prepared in three conditions (microbial concentration reduction, sterilization and hydrogen peroxide). The residual mass was quantified and tested for keratinase production. The most potent enzymatic extract was used in the precipitation technique with salts and organic solvents. The best results of enzymatic activity were obtained using *F. oxysporum*, on the 6thday of fermentation, obtaining 243.25 U mL^–1^ using sterilized swine hair as the substrate. *Aspergillus* sp. showed the highest keratinolytic activity on the 9thday, 113.50 U mL^–1^ using feathers as the substrate. The highest degradation percentage was 59.20% (w/w) in swine hair and the precipitation technique, with relative activities close to 50%. The results are promising for the application of residues and microorganisms in biotechnological processes of economic and environmental interest.

## Graphical Abstract

Schematic diagram of keratinases production using swine hair from agroindustrial residues.

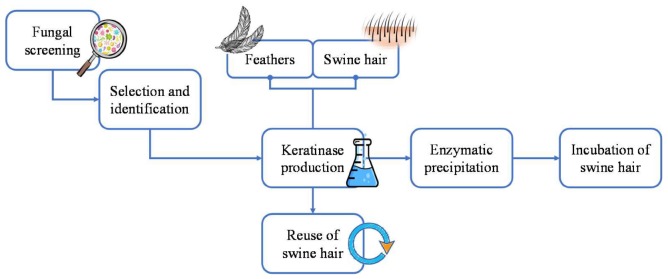

## Introduction

Brazil holds a prominent position in the meat production sector, generating enormous volumes of agroindustrial residues that consist primarily of keratin; these include chicken feathers and swine hair (Pond et al., 1991; Onifade et al., 1998; BAAP, 2018; Freitas et al., 2018). Keratins are complex proteins formed by α-helix and β-sheets structures. The α-keratins are found in vertebrates and have many sulfur conformations; β-keratins are unique to birds and reptiles and generally have smaller structures than α-keratins (Korniłłowicz-Kowalska, 1997; Korniłłowicz-Kowalska and Bohacz, 2011; Wang et al., 2016).

Keratinous wastes are often sent to non-eco-friendly destinations including incinerators and landfills (Agrahari and Wadhwa, 2010; Starón et al., 2017). An alternative that would harness the values of these residues would be to use them to obtain fungi. In this scenario, it is possible to produce proteins with substantial commercial value and at the same time pre-treat the residue with microorganisms (Łaba et al., 2015). Subsequently, the remaining residue can be sent for composting (Choińska-Pulit et al., 2019) or some other fermentation process.

Many fungal species that produce keratinases are found in soils that contain keratin, where the fungi use keratin as a source of food and shelter (Caretta and Piontelli, 1975; Anbu et al., 2008; Ganaie et al., 2010; Bohacz and Korniłłowicz-Kowalska, 2012; Ismail et al., 2012; Kachuei et al., 2012) using surface erosion and radial penetration (Korniłłowicz-Kowalska and Bohacz, 2011).

The role of keratinases is paramount in destabilizing keratin arrangements; nevertheless, synergistic behavior with other functional proteases is also observed (Lopes et al., 2011; Mazotto et al., 2013). The specific activities of these enzymes may be increased using enzymatic concentration techniques such as precipitation. This methodology uses low-cost reagents to separate enzymes from interferents (Sala et al., 2014; Preczeski et al., 2018). Keratinases, both crude and concentrated, have several applications, especially in the environmental area (Cervantes-González et al., 2009; Paul et al., 2014; Bhange et al., 2015; Reddy et al., 2017; Su et al., 2017; Abdel-Fattah et al., 2018; Choińska-Pulit et al., 2019; Kalaikumari et al., 2019).

The production of homemade enzymes is very relevant in the sense that they combat various environmental problems. It is also important to mention that keratinases employ waste as the sole source of nutrients, thereby not competing with the food and energy chains. In the present study, we evaluated strategies for obtaining keratinases produced by fungi using submerged fermentation and two residues as a source of keratin, chicken feathers and swine hair.

## Materials and Methods

### Keratin Substrate

Chicken feathers and swine hair were obtained from a food agroindustry, located in the state of Rio Grande do Sul, Brazil. The material was stored at 4 ± 2°C until use. For use in the fermentation process, the keratin substrates were prepared in the following three conditions (a) Microbial concentration reduction: washing with distilled water, immersion in 70% (v v^–1^) alcohol for 2 h and drying at 70 ± 2°C for 15 h (Călin et al., 2017); (b) Sterilization: washing with distilled water and autoclaving at 1 atm, 121°C for 15 min; and (c) Hydrogen peroxide (H_2_O_2_): 7.50 g of substrate was washing with distilled water and pre-treating with alkaline (pH 11.50) solution 4% (v v^–1^ from 30% stock solution v v^–1^) H_2_O_2_ on an orbital shaker at 150 rpm, 27°C for 3 h (Venturin et al., 2018; De Paris Júnior et al., 2019), followed by immersion in alcohol 70% (v v^–1^) for 2 h and drying at 70 ± 2°C for 15 h. Sterilization and hydrogen peroxide process were applied as pre-treatments aiming at facilitating the access of keratinolytic enzymes to the substrate.

### Fungi Isolation, Selection and Identification

The fungi were isolated from chicken feathers collected in a rural property located in Rio Grande do Sul, Brazil. The fungi collected were incubated on Potato Dextrose Agar (PDA) medium. Four morphologically distinct fungal strains were isolated.

The selection of isolated fungi was performed by inducing keratinase production. To do this, the chicken feathers were cut to 2 cm pieces, washed with distilled water, emerged for 2 h in 70% (v v^–1^) alcohol and dried at 70 ± 2°C for 15 h (Adapted from Călin et al., 2017). Subsequently, 0.10 g chicken feathers were placed on the surface of PDA medium and isolated fungi were inoculated for 7 days at 28°C. The viable spores produced after 7 days of fungi growth were microscopically quantified (production of spores was of about 10^6^ spores. mg^–1^).

The microorganisms were transferred to Erlenmeyer flasks containing 50 mM Tris HCl (pH 7.5) buffer and 10 g L^–1^ clean chicken feathers. Samples were incubated on an orbital shaker at 150 rpm, 28°C for 7 days. Finally, the degradation potential of the substrate was evaluated.

The fungi with the highest degradation potential of the keratin substrate were identified molecularly by the Next Generation Sequencing (NGS) methods (Płoski, 2016).

### Keratinase Production and Enzyme Activity

For submerged fermentation, 10 g L^–1^ of each keratin residue was used as the sole source of C, N, and P, and it was placed in 50 mM Tris HCl (pH 8.5) buffer, and the fungal inoculum was added in the concentration of 10^6^ spores mL^–1^, considering only viable cells. The assays were incubated on an orbital shaker at 150 rpm, 28°C for 9 days (Adapted from Anitha and Palanivelu, 2013; Łaba et al., 2015; Bagewadi et al., 2018).

Keratinolytic activity was quantified in supernatants on days 3, 6, and 9 of fermentation. The solid masses were dried and the substrate degradation was quantified.

For keratinolytic activity, the supernatants obtained from the fermentation processes and the precipitated enzyme extracts were used for the quantification of keratinase activity. The reaction mixture contained 0.013 g keratin azure (Sigma K8500), 3.2 mL 50 mM Tris HCl (pH 8.5) buffer and 0.8 mL enzyme extract and incubated at 50°C for 1 h. Subsequently, 0.8 mL of 10% (v v^–1^) trichloroacetic acid (TCA) was added in assays, and the absorbance was measured (Bressollier et al., 1999). One unit (U) of keratinase activity was defined as the amount of enzyme required to increase an absorbance by 0.01 at 595 nm under assay conditions.

### Enzymatic Precipitation

The assays of enzymatic precipitation were conducted with the enzyme extract from the swine hair fermentative process. Increases in keratinase activity were evaluated. The following salts and solvents were used in various combinations: NaCl, (NH_4_)_2_SO_4_, absolute acetone, and absolute ethanol. The reactions were prepared with 0.5 mol L^–1^ salt concentration and 50% (v v^–1^) solvent concentration and dripped into the enzyme extract at a flow rate of 10 mL min^–1^. The assays were conducted at 4°C. After precipitation, the volumes were centrifuged at 9,000 ×*g* for 20 min at 4°C and the pellets were resuspended in 50 mM Tris HCl (pH 8.5) buffer (Farag and Hassan, 2004; Preczeski et al., 2018). Keratinolytic activity was measured in both supernatants and pellets.

### Application of Enzymatic Extract in Swine

Concentrated enzyme extract activity was evaluated on a pilot-scale using swine hair as an agroindustrial waste. For that, 0.1 g of swine hair and 7 mL of crude and concentrated enzyme extracts were used. Assay reactions were carried out for 90 days at 28°C, and subsequently, keratinolytic activity was measured (Hamiche et al., 2019).

Undegraded swine hair during first submerged fermentation was reused for more two consecutive fermentative processes. In each fermentation, the substrate was prepared by immersion for 2 h in 70% (v v^–1^) alcohol and dried at 70 ± 2°C for 15 h (Călin et al., 2017). The microorganisms were incubated in 50 mM Tris HCl (pH 8.5) buffer and 10 g L^–1^ clean swine hair. Samples remained on an orbital shaker at 150 rpm and 28°C. The keratinolytic activity was quantified at 6 days. The first fermentation was set as 100% of keratinase activity and the subsequent reactions were calculated accordingly.

The quantification of the keratin substrate degradation was calculated considering the dry mass initially (IM) and the dry mass at the end (FM) of the fermentation process, according to Equation 1.

(1) $$D\left(\%\right)=\left(\frac{\text{IM}-\text{FM}}{\text{IM}}\right)*100$$

## Results and Discussion

### Keratinolytic Fungi

Four morphologically distinct microorganisms were isolated from chicken feathers. Two fungi presented potential for keratinase production; these were genetically identified as *Fusarium oxysporum* and *Aspergillus* sp.

Chicken feather degradation was 36.11 and 42.23% (m m^–1^), during 7 days of cultivation, for *F. oxysporum* and *Aspergillus* sp., respectively. To date, there are few reports in the literature of *F. oxysporum* as a potential producer of keratinase enzymes. *Aspergillus* sp. are often found in soil, with keratinolytic potential (Maitig et al., 2018).

### Enzyme Production and Keratin Degradation

*Fusarium oxysporum* showed the highest keratinases production on the 6th day of fermentation, 243.25 and 239.50 U mL^–1^ using as substrate sterile swine hair and prepared with the microbial concentration reduction method, respectively. Substrate degradation in these assays was 59.20 and 41.87% (m.m^–1^) respectively. Using feathers as fermentative substrate, *F. oxysporum* showed the highest activity with the sterilized substrate (149.00 U mL^–1^), followed by hydrogen peroxide treatment (138.00 U mL^–1^), both in six fermentation days, with degradation percentages of 53.98 and 32.78% (m.m^–1^), respectively. *Aspergillus* sp. showed higher enzymatic activity in sterilized swine hair on the 6th day of fermentation and for sterilized chicken feathers on the 9 day, obtaining 112.25 and 113.50 U mL^–1^ and degradations of 34.67 and 39.08% (m.m^–1^), respectively (Table 1).

**TABLE 1:** Keratinolytic activity at various times of submerged fermentation for *Fusarium oxysporum* and *Aspergillus* sp. on chicken feathers and swine hair substrates prepared under various conditions.

		Keratinolytic activity (U mL^–1^)*					
Substrate	Preparation	*Fusarium oxysporum*			*Aspergillus* sp.		
		3 days	6 days	9 days	3 days	6 days	9 days
Chicken feathers	Microbial concentration reduction (a)	21.75	63.75	64.50	7.25	16.00	44.25
	Sterilization (b)	61.00	149.00	110.50	10.50	53.00	113.50
	Hydrogen peroxide (c)	64.00	138.00	56.50	8.50	22.50	21.75
Swine hair	Microbial concentration reduction (a)	198.75	239.50	197.50	0.00	5.25	5.75
	Sterilization (b)	178.75	243.25	172.75	58.50	112.25	48.75
	Hydrogen peroxide (c)	69.25	109.50	74.50	36.75	68.75	76.50

*All standard deviation were lower than 10%.

The results have enormous potential for real-scale application, considering the application in swine hair using native fungi, without adding genetic improvements and, considering results of keratinase production from real substrates of the livestock industry. Mazotto et al. (2013) used chicken feather powder as a substrate, obtaining keratinolytic activity of 60 and 172.7 U mL^–1^ at 7 days in submerged fermentation and solid-state fermentation, respectively, using mutant strains of *Aspergillus niger*. Lopes et al. (2011) found approximately 0.7 U mL^–1^ of keratinolytic activity at 72 h of submerged fermentation using *A. niger* and swine hair without some pre-treatment, using azocasein as the substrate. Kalaikumari et al. (2019) used Plackett-Burman design for *Bacillus paralicheniformis* MKU3 keratinases production, using chicken feathers as fermentative substrate, obtaining 155 U mL^–1^ on the 4th day of processing.

Studies show that the genera *Aspergillus* and *Fusarium* are potentially keratinolytic fungal species, acting effectively on keratin substrates (Lopes et al., 2011; Kannahi and Ancy, 2012; Mazotto et al., 2013). Generally, keratin degradation by filamentous fungi occurs first by producing of hyphae that growing around the keratin strand, compromising the integrity of the strand by surface erosion and lifting of cuticular scales (Călin et al., 2017).

This process of fungal degradation is also the result of enzymatic acting on the keratin structure. The complex mechanism of keratin degradation involves the reduction of disulfide bridges of the keratin structure mediated by disulfide reductases, releasing thiol groups in the form of cysteine and S-sulfocysteine. This process alters the structural conformation of keratin, enabling proteolysis by endo and exoproteolytic enzymes such as keratinases, resulting in the release of soluble peptides and amino acids (Yamamura et al., 2002). Enzyme yields by fungi depend on substrate composition, because keratinous structures are composed not only of keratin (Monod, 2008).

The highest enzymatic activities for *F. oxysporum*, 243 and 239 U mL^–1^, were obtained when swine hair was used as the substrate, even though the fungus was isolated from chicken feathers. It is therefore important to stress that different structures form these substrates and that the synergism between enzymes and the action on keratin structure can differ (Lopes et al., 2011; Mazotto et al., 2013). Bird feathers have about 32 keratin residues in the β-sheet matrix organized into structures of about 100 residues. The remaining matrix consist of other compounds with hydrophobic characteristics essential for material stability (Fraser and Parry, 2008). The α-helix molecular structure is found in mammalian hair, nails, and horns (Wang et al., 2015). It has been suggested that enzymatic action in swine hair is mediated by an enzymatic pool, including at least three enzymes that synergistically degrade keratin: endoprotease, exoprotease, oligopeptidase (all of which are produced after sulpholysis), and keratinase belonging to the exoprotease family (Huang et al., 2015; Lange et al., 2016).

Promising results in terms of enzyme production were also obtained for both substrates in relation to the two fungi studied, *F. oxysporum* with 149 and 138 U mL^–1^ in chicken feathers, and *Aspergillus* sp. with 113.5 and 112.25 U mL^–1^ for chicken feathers and swine hair, respectively. These findings suggest that structural and substrate preparation differences induced the variations in enzyme production, and this may be related to the use of waste as substrates.

The substantial amounts of enzymatic activity observed for *F. oxysporum* in swine hair prepared by microbial concentration reduction makes this process promising. This is because most fermentative processes depend on high pressure and temperature sterilization, making these processes dependent on complex systems, generating high costs (Adler et al., 2018). The ability of the fungus to produce keratinases using chicken feather and swine hair as the sole source of carbon and nitrogen makes the process simple and easily scalable.

Most data in the literature consider only β-keratin. The present study differs from the others with respect to our findings of activities higher than 200 U mL^–1^ using a substrate with α-helix structure (swine hair), suggesting a valuable use for this residue that otherwise is considered an industrial problem.

Enzymes produced by *F. oxysporum* accessed the complex structure of the keratin substrate without chemical or physical disruption prior to the inoculation of the microorganism. Therefore, we used the microbial concentration reduction method to prepare substrate.

### Screening of Precipitation and Incubation of Swine Hair

**FIGURE 1:**
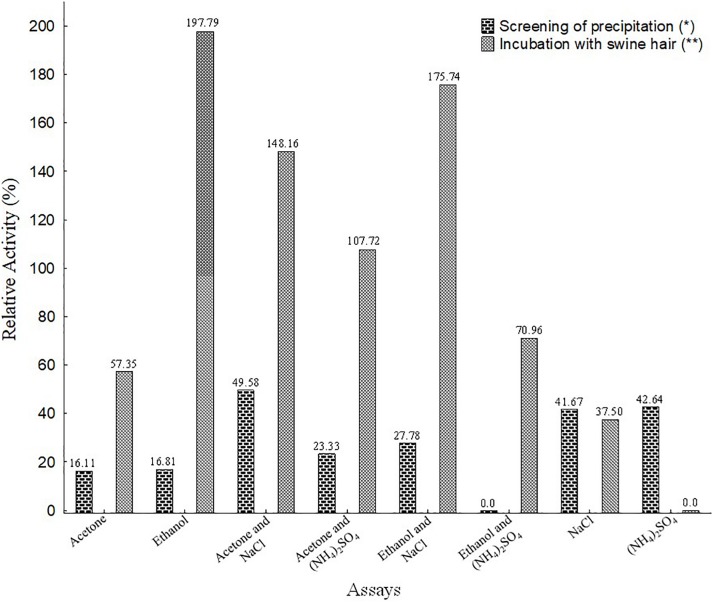
Relative activity of enzymatic precipitation screening and 90 days after swine hair incubation with concentrated enzyme extracts. Crude *180 and **272 U mL^–1^ – Relative activity of the crude enzyme was 0.0%.

Enzyme extract of keratinases produced by *F. oxysporum* with swine hair prepared by the microbial concentration reduction method was performed by enzymatic precipitation using ammonium sulfate, sodium chloride, acetone, and ethanol. The highest relative activity was obtained by acetone and sodium chloride addition (49.58%), followed by ammonium sulfate (42.64%) and sodium chloride (41.67%). All assays had enzymatic yields between 47 and 74%. The results were obtained in precipitates (pellets), suggesting that the process is efficient in precipitating proteins of interest by separating them from enzymatic impurities (Figure 1).

Several studies have used ammonium sulfate as a precipitating agent for keratinases (Farag and Hassan, 2004; Kim, 2007; Anbu et al., 2008; Rahayu et al., 2012; Hamiche et al., 2019). Rahayu et al. (2012) obtained 0.118 U mL^–1^ of keratinases activity by precipitating 50% ammonium sulfate (w v^–1^), reaching 3.38% enzymatic yield. Farag and Hassan (2004) precipitated *Aspergillus oryzae* keratinases, using ammonium sulfate, acetone or ethanol at 50%. They recorded 103.93, 109.18, and 107.85% relative activities, respectively. In the current study, we found higher enzymatic increments than did other studies that also used precipitated keratinases with salts and organic solvents.

Salts and organic solvents in the enzymatic precipitation processes promote boost the ionic strength of the system and reduce the dielectric constant, thereby promoting protein-protein interactions that stand out from water-protein interactions, resulting in precipitated protein aggregates (McPherson, 2004; Zhang et al., 2007; Kramer et al., 2012; Möller et al., 2012).

The majority of the 90-day incubation assays of concentrated extracts showed increased keratinase activity using various combinations of precipitating compounds. These results suggest that the enzymatic concentration process effectively reduces impurities, allowing greater use of the enzyme active sites. Generally, enzymes are denatured or have their activities decreased during prolonged contact with organic solvents; however, in the present study, the enzyme showed stability and activation during prolonged incubation periods. This fact corroborates the findings of Rahman et al. (2006), who observed increases in alkaline protease activity in the presence of organic solvents.

These results show special importance in the present study, as it was proved that a single precipitating agent reached high values of keratinolytic activity with enzymatic extract produced with agro-industry residues, reducing the costs of the precipitation process.

### Swine Hair Reuse

The reuse of swine hair for keratinase production was evaluated during three consecutive batch fermentations. Enzymatic production of the third reuse fermentation maintained 75% of the initial enzymatic activity. Studies have shown that it is possible to recycle in the course of biotechnological processes, maintaining at the end a percentage of production of compounds of interest very similar to the first production. In this study, the hypothesis for the reduction of keratinases production during fermentative cycles was based on the composition of the keratin structure. Keratinases are produced by fungi after sulpholysis performed by disulfide reductases. The more often the waste is reused, the fewer bonds that need keratinases to be disrupted, creating a greater need to produce other proteases that are not of interest to the study (Yamamura et al., 2002; Babaki et al., 2016; Andrade et al., 2017).

This result of swine hair reuse is important for the development of industrial-scale processes. Substrates can be reused without significant loss of enzymatic activity, without the need of high pressure and temperature sterilization, without supplementation of carbon sources and nitrogen and with the use of an agro-industrial residue possessing high polluting potential.

## Conclusion

*Fusarium oxysporum* and *Aspergillus* sp. isolated from chicken feathers presented high keratinase production. *F. oxysporum* produced activity of 239 U mL^–1^ using swine hair as the sole source of carbon and nitrogen. The enzymatic precipitation technique with various organic solvents increased the enzymatic activity, demonstrating that enzymatic precipitation effectively removed interferents, while the enzyme remained stable and active in the presence of the compounds. In substrate reuse studies, the production potential of keratinases was maintained at 75%, demonstrating the potential for industrial batch production enzymatic processes.

## Data Availability Statement

The datasets generated for this study are available on request to the corresponding author.

## Author Contributions

KP, CD, FC, SK, TS, AC, JZ, CB, FS, and BV: experimental procedures, results discussion, and data treatment. GF and HT: research coordinators.

## Conflict of Interest

The authors declare that the research was conducted in the absence of any commercial or financial relationships that could be construed as a potential conflict of interest.
